# A prospective external validation of the GRade, Age, Nodes and Tumor score in the ECOG-ACRIN EA8143 PROSPER trial

**DOI:** 10.1093/oncolo/oyag041

**Published:** 2026-02-16

**Authors:** Sebastiano Buti, Se-Eun Kim, Michele Maffezzoli, Mohamad E Allaf, Giulia Claire Giudice, Giuseppe L Banna, Satoru Taguchi, Naomi B Haas, Michael A Carducci

**Affiliations:** Medicine and Surgery Department, University of Parma, Parma, 43126, Italy; Medical Oncology Unit, University Hospital of Parma, Parma, 43126, Italy; Department of Data Science, Dana-Farber Cancer Institute/ECOG-ACRIN Biostatistics Center, Boston, MA, 02215, United States; Medicine and Surgery Department, University of Parma, Parma, 43126, Italy; Department of Oncology Unit, Portsmouth University Hospital NHS Trust, Portsmouth, PO6 3LY, United Kingdom; Department of Urology, The James Buchanan Brady Urological Institute, Johns Hopkins University School of Medicine, Baltimore, MD, 21287, United States; Medical Oncology Unit, Azienda USL-IRCCS di Reggio Emilia, Reggio Emilia, 42122, Italy; Department of Oncology Unit, Portsmouth University Hospital NHS Trust, Portsmouth, PO6 3LY, United Kingdom; Faculty of Science and Health, School of Pharmacy and Biomedical Sciences, University of Portsmouth, Portsmouth, PO1 2DT, United Kingdom; Department of Urology, Graduate School of Medicine, The University of Tokyo, Tokyo, 113-8655, Japan; Department of Medicine, University of Pennsylvania/Abramson Cancer Center, Philadelphia, PA, 19104, United States; Perelman Center for Advanced Medicine, University of Pennsylvania, Philadelphia, PA, 19104, United States; Sidney Kimmel Cancer Center, Johns Hopkins University, Baltimore, MD, 21287, United States

**Keywords:** GRANT, immunotherapy, neoadjuvant, nivolumab, perioperative, prognosis, PROSPER, renal cancer, surgery

## Abstract

**Background:**

The GRade, Age, Nodes and Tumor (GRANT) score is one of the prognostic models recommended by international guidelines to refine recurrence risk stratification in patients with surgically treated renal cell carcinoma (RCC) and integrates age, tumor size, grade, and nodal status. In this study, we aimed to validate the GRANT score within the ECOG-ACRIN EA8143 PROSPER prospective trial.

**Methods:**

We conducted a validation analysis of the GRANT score within the phase III randomized EA8143 PROSPER study of perioperative nivolumab in surgically treated RCC. Patients were classified into 2 risk groups, favorable (0-1 risk factors) versus unfavorable (2-4 risk factors). Relapse-free survival (RFS) and overall survival (OS) were estimated using the Kaplan–Meier method. Model discrimination were evaluated using Harrell’s C-index.

**Results:**

Among 714 patients included, 58.3% were favorable and 41.7% unfavorable based on the GRANT score. Patients in the favorable group had a significantly longer median RFS (61.1 vs. 36.9 months; hazard ratio [HR]: 0.36, 95% confidence interval [CI]: 0.27-0.48, *P *< .001) and OS (median not reached, HR: 0.25, 95% CI: 0.15-0.42, *P *< .001) compared to patients in the unfavorable group. The c-index was 0.63 and 0.66 for RFS and OS, respectively. A better prognostic performance was observed among nonclear cell RCC for both RFS (HR: 0.13, 95% CI: 0.05-0.33, *P *< .001; c-index: 0.74) and OS (HR: 0.14, 95% CI: 0.04-0.50, *P *< .001; c-index 0.74).

**Conclusions:**

The GRANT score was prospectively validated in the PROSPER study, demonstrating prognostic value for both RFS and OS, especially in nonclear RCC, further supporting its use in clinical practice. Clinical trial registration number: ClinicalTrials.gov, NCT03055013.

Implications for Practice This is the first study to validate the GRade, Age, Nodes and Tumor (GRANT) prognostic score in a large prospective trial of patients with renal cell carcinoma (RCC) treated with surgery and immunotherapy-based perioperative treatment. The score accurately predicted relapse-free and overall survival, especially in nonclear cell RCC.

## Introduction

Approximately 20%-40% of patients with renal cell carcinoma (RCC) experience disease recurrence after curative surgery.^1^ Several factors play a role in determining the risk of recurrence, including tumor size and stage, nuclear grade, tumor necrosis, and sarcomatoid differentiation.^2^ Various prognostic models incorporating these variables, including the GRade, Age, Nodes, and Tumor (GRANT) score, have been developed to predict patient prognosis (Table S1).^3–6^ In addition to these models, 2 nomograms are commonly used in clinical practice: the Leibovich 2018 and the ASSURE nomogram.^7^^,^^8^ These models are recommended by international guidelines to guide patient counselling and personalize surveillance.^9^

The GRANT score is a simple prognostic model that stratifies patients into risk categories based on Fuhrman’s grade, patient’s age, pathological tumor size and nodal status.^6^ The score was initially developed within a randomized phase III adjuvant trial investigating low-dose IL-2 and interferon-α versus observation, demonstrating prognostic and potential predictive value.^10^ Given these promising results, the GRANT score was validated in a larger population within the ASSURE trial (ECOG-ACRIN E2805) with adjuvant sunitinib or sorafenib versus placebo, showing good prognostic prediction for both overall survival (OS) and disease-free survival (DFS).^6^ Of note, both the development and validation occurred in prospective trials, further reinforcing the reliability of the score. Eventually, the score was externally validated in 2019, on a huge population of 73 217 patients from the Surveillance, Epidemiology, and End Results (SEER) database.^11^

In this analysis, we aimed to validate the GRANT score in the prospective RCC population of the phase III randomized PROSPER EA8143 trial, which evaluated the efficacy of perioperative nivolumab *vs* surgery alone in patients with high-risk RCC.^12^ This represents the first application of a prognostic model in a study evaluating immune checkpoint inhibitor (ICI)-based perioperative therapy. Our objective was to assess the score’s ability to stratify patients into distinct prognostic risk groups.

## Methods

We conducted a validation study of the GRANT score within the PROSPER EA8143 trial, an open-label, randomized, multicenter, phase III trial. Eligible patients were aged 18 years or older, with an ECOG PS of 0-1, diagnosed with previously untreated clinical stage T2 or higher RCC or any clinical T stage and node-positive RCC of any histology planned for radical or partial nephrectomy. Patients were randomly assigned (1:1) to receive nivolumab plus surgery or surgery only. Nivolumab was given as a single preoperative dose 1-4 weeks before surgery, followed by 9 months of postoperative treatment in the investigational group. The control group underwent surveillance. The detailed list of inclusion and exclusion criteria, and additional details of the PROSPER study can be found in the original paper.^12^

Data regarding recurrence, death, age, histology (clear cell and nonclear cell including papillary, chromophobe, mixed histology, unclassified and missing), pathological T- and N-stage (according to TNM 8th edition), Fuhrman grade (G1, G2, G3, and G4) and treatment arm were extracted. These parameters were used to calculate the GRANT score and patients were assigned one point for each of the following: age >60 years, tumor grade >2, pathologic T-stage ≥ pT3b, and pathologic N-stage other than N0 or NX, resulting in a score range of 0-4.^6^ Patients with score 0-1 were classified as favorable, while patients with score 2-4 were classified as unfavorable.^6^

Relapse-free survival (RFS) was defined as the time from randomization to disease recurrence or death from any cause. OS was intended as the time from randomization to death from any cause. Patients without recurrence or alive at the time of analysis were censored at the date of last disease evaluation or last known alive date, respectively. Kaplan–Meier method was used to estimate RFS and OS curves, and the log-rank test applied to assess statistically significant differences between subgroups. The proportional hazards assumption was assessed using goodness-of-fit test. Univariable and multivariable Cox proportional hazards model were used to estimate the association between GRANT groups and survival outcomes, incorporating different subgroups (treatment arm, histology group). Interaction terms were used to assess the relationship between GRANT groups and survival outcomes. Model discrimination were evaluated using Harrell’s C-index.^13^ DCA plots were generated to determine the net benefit of the score.^14^ The treat-all strategy was defined as treating all patients after nephrectomy, while the treat-none strategy assumed no patient would receive adjuvant treatment.

Results were expressed as hazard ratio (HR), 95% confidence intervals (95% CIs), and *P*-values, with a level of two-sided significance set at *P *< .05. The software R (version 4.4.2) were used to perform all the analyses.

## Results

### Patient characteristics

Out of the 819 patients assessed for eligibility, 105 patients were excluded due to missing variables. A total of 714 patients were included in the final analysis (Figure S1, see online supplementary material for a color version of this figure).

General characteristics of the population were summarized in Table 1. The median age was 60 years (range: 23-88). 408 patients (57.1%) had pathological stage T3a, while only 65 (9.1%) had pathological lymph node involvement. A tumor grade ≥ 3 was reported in 466 patients (68.6%), with 180 patients (26.5%) having G4 tumor. Clear cell RCC was the more prevalent histology, presenting in 606 patients (85.5%). A total of 342/714 patients (47.9%) were randomized to the nivolumab plus surgery arm. Based on the GRANT score, 416 patients (58.3%) of patients were classified as favorable risk (0-1 risk factors), while 298 (41.7%) were in the unfavorable risk group (2-4 risk factors).

**Table 1:** General characteristics of the population.

	Overall (*N* = 714)
**Age**	
** Median**	60.0
** Range**	23.0-88.0
**Pathological T-stage**	
** T1a**	12 (1.7%)
** T1b**	61 (8.5%)
** T2a**	103 (14.4%)
** T2b**	54 (7.6%)
** T3a**	408 (57.1%)
** T3b**	42 (5.9%)
** T3c**	6 (0.8%)
** T3x**	5 (0.7%)
** T4**	23 (3.2%)
**Pathological N-stage**	
** Nx**	356 (49.9%)
** N0**	293 (41.0%)
** N1**	65 (9.1%)
**Fuhrman grade**	
** 1**	22 (3.2%)
** 2**	191 (28.1%)
** 3**	286 (42.1%)
** 4**	180 (26.5%)
** Missing**	35
**Renal cell carcinoma (RCC) subtype**	
** Clear cell RCC**	606 (85.5%)
** Nonclear cell RCC**	103 (14.5%)
** Missing**	5
**Treatment arm**	
** Nivolumab plus surgery arm**	342 (47.9%)
** Surgery only arm**	372 (52.1%)
**Number of unfavorable factors**	
** 0**	132 (18.5%)
** 1**	284 (39.8%)
** 2**	240 (33.6%)
** 3**	48 (6.7%)
** 4**	10 (1.4%)
**GRANT risk group**	
** Favorable**	416 (58.3%)
** Unfavorable**	298 (41.7%)

Pathological stage is according to TNM 8th edition. GRade, Age, Nodes and Tumor (GRANT) favorable group: score 0-1; GRANT unfavorable group: score 2-4 (1 point each for: age >60 years, tumor grade >2, pT-stage ≥pT3b and pN1).

### Recurrence-free survival analysis

**Figure 1:**
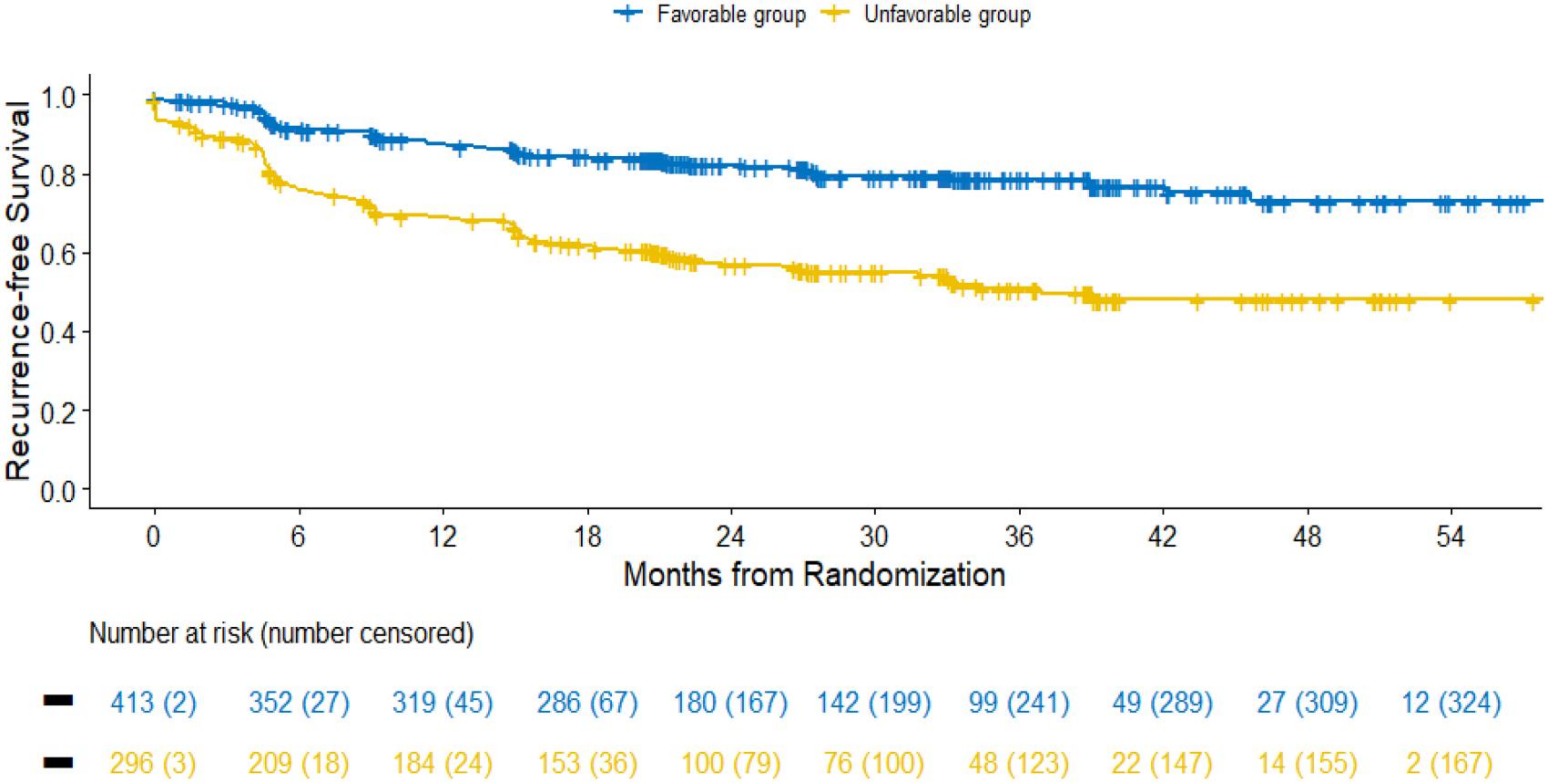
Kaplan–Meier curves of recurrence-free survival in the GRade, Age, Nodes and Tumor (GRANT) groups. GRANT favorable group: score 0-1; GRANT unfavorable group: score 2-4 (1 point each for: age >60 years, tumor grade >2, pT-stage ≥pT3b and pN1).

RFS was significantly different between favorable and unfavorable GRANT groups: median RFS was 61.1 months (95% CI: NA-NA) and 36.9 months (95% CI: 26.8-NA) respectively, (HR: 0.36, 95% CI: 0.27-0.48, *P *< .001). At predefined time points, 2- and 4-year RFS were 82.3% (95% CI: 78.4-86.3) and 73.0% (95% CI: 66.3-80.4), respectively, in the favorable group, compared to 56.8% (95% CI: 51.0-63.2) and 48.1% (95% CI: 41.4-56.0), respectively, in the unfavorable group. The model’s c-index was 0.63 (95% CI: 0.60-0.66) (Table 2 and Figure 1). The DCA plot for RFS showed that the GRANT score had a consistent net benefit for threshold probabilities approximately between 25% and 50% (Figure S2, see online supplementary material for a color version of this figure).

**Table 2:** Kaplan–Meier estimates of recurrence-free survival in the GRANT groups.

GRANT risk group	Number of event/total	2-year RFS [95% CI] (%)	4-year RFS [95% CI] (%)	Median [95% CI] (months)	HR [95% CI]	*P*-value	c-index [95% CI]
**Favorable**	78/413	82.3 [78.4-86.3]	73.0 [66.3-80.4]	61.1 [NA-NA]	0.36 [0.27-0.48]	**<.001**	0.63 [0.60-0.66]
**Unfavorable**	127/296	56.8 [51.0-63.2]	48.1 [41.4-56.0]	36.9 [26.8-NA]			

GRade, Age, Nodes and Tumor (GRANT) favorable group: score 0-1; GRANT unfavorable group: score 2-4 (1 point each for: age >60 years, tumor grade >2, pT-stage ≥pT3b and pN1). Bold values indicate statistical significance.

Abbreviations: CI, confidence interval; HR, hazard ratio.

When stratifying by treatment arm, no significant differences in RFS were observed among GRANT risk groups. In the favorable group, median RFS was 61.1 months (95% CI: NA-NA) in the nivolumab arm compared to not reached (95% CI: NA-NA) in the surgery only arm (HR: 0.66, 95% CI: 0.42-1.05, *P *= .075). In the unfavorable- group, median RFS was 39.1 months (95% CI: 27.3-NA) in the experimental arm compared to 36.9 months (95% CI: 21.0-NA) in the control group (HR 0.90, 95% CI: 0.64-1.28, *P *= .56). The interaction test between GRANT group and treatment arm was not significant (*P *= .33; Table S2 and Figure S3, see online supplementary material for a color version of this figure).

The stratification by histology subtype revealed significant differences in RFS among GRANT groups. In patients with clear cell histology, the median RFS was 61.1 months (95% CI: NA-NA) and not reached (95% CI: 31.8-NA) in the favorable and unfavorable group, respectively (HR 0.42, 95% CI: 0.31-0.57, *P *< .001). The c-index was 0.61 (95% CI: 0.57-0.65). Among patients with nonclear cell RCC (non-ccRCC), the favorable group had a not reached median RFS, while the unfavorable group had a median RFS of 23.7 months (95% CI: 9.3-NA), HR 0.13 (95% CI: 0.05-0.33, *P *< .001; Table 3). The c-index was 0.74 (95% CI: 0.66-0.81). Additionally, a significant interaction effect between GRANT group and histology subtype was observed (*P *= .012; Table 4 and Figure S4, see online supplementary material for a color version of this figure).

**Table 3:** Subgroup univariable Cox regression models for recurrence-free survival by GRANT group and histology subtype, with interaction analysis (*n* = 709).

Histology	GRANT risk group	Number of event/total	Median [95% CI] (months)	HR [95% CI] (favorable/unfavorable)	*P*-value	c-index [95% CI]
**Clear cell histology (*n* = 606)**	Favorable	72/353	61.1 [NA-NA]	0.42 [0.31-0.57]	<.001	0.61 [0.57-0.65]
	Unfavorable	104/253	NA [31.8-NA]			
**Nonclear cell histology (*n* = 103)**	Favorable	6/60	NA [NA-NA]	0.13 [0.05-0.33]	<.001	0.74 [0.66-0.81]
	Unfavorable	23/43	23.7 [9.3-NA]			

GRade, Age, Nodes and Tumor (GRANT) favorable group: score 0-1; GRANT unfavorable group: score 2-4 (1 point each for: age >60 years, tumor grade >2, pT-stage ≥pT3b and pN1).

Abbreviations: CI, confidence interval; HR, hazard ratio.

**Table 4:** Interaction analysis for recurrence-free survival between GRANT group and histology subtype.

Variable	Comparison	HR [95% CI]	*P*-value
**GRANT risk group**	Favorable vs. unfavorable (ref)	0.13 [0.05-0.31]	<**.001**
**Histology subtype**	Clear cell RCC versus nonclear cell RCC (ref)	0.66 [0.42-1.03]	.068
**Interaction test**	GRANT risk group × histology subtype	3.38 [1.31-8.73]	**.012**

GRade, Age, Nodes and Tumor (GRANT) favorable group: score 0-1; GRANT unfavorable group: score 2-4 (1 point each for: age >60 years, tumor grade >2, pT-stage ≥pT3b and pN1). Bold values indicate statistical significance.

Abbreviations: CI, confidence interval; HR, hazard ratio.

In patients with clear cell histology, the favorable group showed 2- and 3-year RFS of 80.9% (95% CI: 76.6-85.4) and 77.2% (95% CI: 72.3-82.4), respectively, compared to 58.5% (95% CI: 52.3-65.3) and 53.0% (95% CI: 46.2-60.8), respectively, in the unfavorable group. Among patients with nonclear cell tumors, 2- and 3-year RFS were 90.3% (95% CI: 82.3-99.0) and 86.9% (95% CI: 77.2-97.9), respectively, in the favorable group, and 46.8% (95% CI: 33.1-66.2) and 38.2% (95% CI: 24.3-59.8), respectively, in the unfavorable group (Table S3).

The DCA plot for RFS stratified by histology showed that the GRANT score provided a consistent net benefit for threshold probabilities approximately between 20% and 45% in ccRCC, and across a broader range, approximately between 15% and 60%, in non-ccRCC (Figure S5, see online supplementary material for a color version of this figure).

### Overall survival

**Figure 2:**
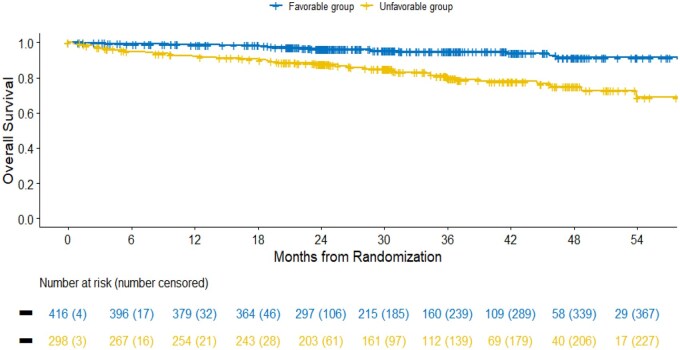
Kaplan–Meier curves of overall survival in the GRade, Age, Nodes and Tumor (GRANT) groups. GRANT favorable group: score 0-1; GRANT unfavorable group: score 2-4 (1 point each for: age >60 years, tumor grade >2, pT-stage pT3b and pN1).

OS was significantly different between GRANT risk groups. The median OS was not reached in either risk groups, but patients in the favorable group had a significantly lower risk of death compared to those in the unfavorable group (HR: 0.25, 95% CI: 0.15-0.42, *P *< .001; Table 5). At predefined time points, 2- and 4-year OS were 96.5% (95% CI: 94.7-98.4) and 91.4% (95% CI: 87.0-96.0), respectively, in the favorable group, compared to 87.8% (95% CI: 84.0-91.7) and 75.0% (95% CI: 68.5-82.2), respectively, in the unfavorable group. The c-index was 0.66 (95% CI: 0.61-0.72; Table 5 and Figure 2). The DCA plot for OS demonstrated that the GRANT score had a consistent net benefit for threshold probabilities approximately between 10% and 25% (Figure S6, see online supplementary material for a color version of this figure).

**Table 5:** Kaplan–Meier estimates of overall survival in the GRANT groups.

GRANT risk group	Number of event/total	2-year OS [95% CI] (%)	4-year OS [95% CI] (%)	Median [95% CI] (months)	HR [95% CI]	*P*-value	c-index [95% CI]
**Favorable**	20/416	96.5 [94.7-98.4]	91.4 [87.0-96.0]	NA [NA-NA]	0.25 [0.15-0.42]	<.001	0.66 [0.61-0.72]
**Unfavorable**	55/298	87.8 [84.0-91.7]	75.0 [68.5-82.2]	NA [59.5-NA]			

GRade, Age, Nodes and Tumor (GRANT) favorable group: score 0-1; GRANT unfavorable group: score 2-4 (1 point each for: age >60 years, tumor grade >2, pT-stage ≥pT3b and pN1).

Abbreviations: CI, confidence interval; HR, hazard ratio.

When stratifying by treatment arm, no significant differences in OS were observed between GRANT risk groups. In both the favorable and unfavorable groups, median OS was not reached in either the nivolumab plus surgery or the surgery-only arms (favorable HR 1.00, 95% CI: 0.42-2.41, *P* > .99; unfavorable HR 1.20, 95% CI: 0.71-2.04, *P* = .50). The interaction test between GRANT group and treatment arm was not significant (*P *= .73; Table S4 and Figure S7, see online supplementary material for a color version of this figure).

The stratification by histology subtype revealed significant differences in OS among GRANT risk groups. The median OS was not reached in both GRANT risk groups among patients with clear-cell histology, but patients in the favorable group exhibited a significantly lower risk of death (HR 0.29, 95% CI: 0.16-0.50, *P *< .001). The c-index was 0.64 (95% CI: 0.58-0.71). Similarly, in the nonclear cell histology, the median OS was not reached in both GRANT groups, but the favorable one had a significantly lower risk of death (HR 0.14, 95% CI: 0.04-0.50, *P *< .001). The c-index was 0.74 (95% CI: 0.65-0.83). However, no significant interaction effect was observed between GRANT group and histology subtype (*P *= .32; Table S5 and Figure S8, see online supplementary material for a color version of this figure).

The 2- and 3-year OS for patients with clear and nonclear cell histology across GRANT risk groups were summarized in Table S6. The DCA plot for OS stratified by histology showed that the GRANT score had a consistent net benefit for threshold probabilities approximately between 10% and 20% in ccRCC, and across a broader range, approximately between 15% and 45%, in non-ccRCC (Figure S9, see online supplementary material for a color version of this figure).

## Discussion

In this study we validated the GRANT prognostic score within the PROSPER EA8143 trial, a phase III prospective randomized trial, which evaluated the efficacy of perioperative nivolumab compared to surgery alone in patients with surgically treated RCC.^12^ By distinguishing between favorable (0-1 risk factors) and unfavorable (2-4 risk factors) groups, the GRANT score demonstrated prognostic value for both RFS and OS, as evidenced by statistically significant differences in survival outcomes (*P *< .001). Despite modest discriminative performance (c-index: 0.63 for RFS and 0.66 for OS), DCA plots showed a consistent net benefit within clinically relevant ranges of threshold probabilities, supporting potential clinical utility, and the score remains easily applicable in routine practice. Our results are consistent with those reported in the development and validation cohorts of the GRANT score, with comparable RFS, OS and c-indices.^6^^,^^10^

While already validated in a prospective cohort in the VEGF-TKI era^6^ and in different retrospective cohorts before the checkpoint inhibitor era,^11^^,^^15^ this study offers the first validation in a recent prospective cohort of patients treated with perioperative ICI. However, in the PROSPER EA8143 trial perioperative nivolumab failed to improve RFS and OS. As expected from a negative study, no significant interaction was found between the score and the treatment arm. Therefore, the GRANT score does not retain any predictive value as it is not able to identify those patients who could benefit from perioperative immunotherapy. The question of how to better identify patients for perioperative or adjuvant therapy, and which prognostic model to use, remains open and still needs to be addressed. It should be noted that some GRANT components (grade, N-stage, and T-stage) may have been downgraded by pre-operative nivolumab, potentially impacting recurrence risk and model’s discriminatory performance. Specifically, any treatment-related reduction in recurrence risk following perioperative nivolumab may have attenuated the prognostic accuracy of the GRANT score. If nivolumab exerted some effect on recurrence risk in ccRCC, this could have reduced the discriminatory ability of the score in this subgroup, resulting in a lower c-index. In contrast, a limited effect of nivolumab on recurrence risk in patients with non-ccRCC may have led to a higher c-index in this population. This represents the major limitation of the present study, complicating the interpretation of a purely clinicopathologic prognostic model and potentially underestimating its true prognostic performance after nephrectomy.

The GRANT score predicted RFS and OS in both clear cell and nonclear cell histology, demonstrating lower HR and higher *c*-indices among patients with nonclear cell tumor. Consistent with these findings, a better prognostic accuracy for OS in non-ccRCC (predominantly papillary) had already been reported in both the ASSURE- and SEER-based validations.^6^^,^^11^ Similar results were observed in a recent validation study in a population with surgically treated papillary RCC.^16^ In the current study, the interaction between the GRANT score and the histology subtype was also significant for RFS, meaning that the score may have a better prognostic accuracy in non-ccRCC. While not significant, a similar trend was also observed for OS. This could be due to the heterogeneity of nonclear cell population: some sub-histology (e.g. chromophobe) may exhibit a more favorable prognosis, while others may have worse survival outcomes. However, it should be acknowledged that the non-ccRCC population represented only 14.4% of the cohort (103 patients). Unfortunately, a further sub-classification of nonclear cell histology was not possible due to paucity of numbers and may require further validation studies within pre-specified populations. The prognostic performance of the score and the consistent net benefit observed in the DCA plots in patients with non-ccRCC, highlight its potential clinical utility in this population, deserving future investigation.

The score is easy to use in clinical practice, based on readily available data, and exhibited comparable performances compared to other validated and more complex prognostic models.^17^ The validation in a large recent prospective trial further improves the strength of the score. Moreover, this represents the first validation of a prognostic model in a study evaluating ICI-based perioperative therapy. Although it is unlikely to change clinical practice, the GRANT score provides an additional tool for prognostic stratification that may support clinicians in tailoring surveillance strategies between favorable and unfavorable groups, particularly in the non-ccRCC population where its prognostic value appeared more significant.

Despite these advantages, there are some limitations. The exclusion of 105 patients (12.8%) due to missing variables may introduce selection bias. Nonetheless, the final cohort of 714 patients still remains representative. It should be noted that the PROSPER trial enrolled only patients with high-risk features (T2+ or N+ disease), thus not fully capturing the full spectrum of patients undergoing surgery for RCC, eventually reducing the generalizability of our findings. Interestingly, the GRANT score was able to further stratify this population by identifying a favorable-risk subgroup. The GRANT score relies on parameters like Fuhrman grade and TNM classification, which may not be consistent across rare histology or evolving classification systems. This could limit its broader applicability. However, a recent study has demonstrated a significant correlation between the Fuhrman and WHO/ISUP grading systems, suggesting that both grading systems are effective in reflecting tumor biology.^18^

In the PROSPER EA8143 trial the median follow-up was approximately 30 months which may not fully capture long-term clinical outcomes and could lead to an underestimation of late events, (whereas this analysis was performed at a later date). Additionally, study’s negative overall results may have influenced the performance of the score.

Eventually, the lack of additional data points limited further insights. The integration of novel biomarkers (e.g. circulating-tumor DNA and KIM-1), advanced imaging techniques, and artificial intelligence (AI) holds promise for more accurate prognostic assessment in patients with RCC. In particular, circulating biomarkers, including KIM-1, can offer a more sensitive and dynamic assessment of recurrence, with the potential to detect minimal residual disease and reduce the need for radiological imaging. The integration of advanced imaging techniques and AI can extract hidden prognostic information from routine radiological and clinical data, reflecting tumor heterogeneity and evolution. The integration of these data may enable more accurate risk stratification, improve patient selection for perioperative or adjuvant therapies, and ultimately reduce both overtreatment and undertreatment, although these approaches may be more expensive, difficult to apply in daily clinical practice and frequently lack validation.^19–23^ Future research should focus on developing integrated models that can incorporate emerging prognostic factors, while accounting for applicability in clinical practice.

## Conclusion

The GRANT score showed prognostic value for RFS and OS in this recent prospective cohort of patients undergoing surgery alone or perioperative nivolumab within the PROSPER EA8143 trial, despite lacking predictive value. These findings support the role of the GRANT score in risk stratification, particularly in nonclear cell histology, where its prognostic value was more significant. Its strength relies on easiness of use but may require the addition of emerging biomarkers to improve its discriminative ability.

## Supplementary Material

oyag041_Supplementary_Data

## Data Availability

Data are available for bona fide researchers who request it from the authors.
