# Supplementary material for: A prospective external validation of the GRade, Age, Nodes and Tumor score in the ECOG-ACRIN EA8143 PROSPER trial
Source: Oncologist. 2026 Feb 16;31(4):oyag041. doi: 10.1093/oncolo/oyag041 (PMC12978424; doi:10.1093/oncolo/oyag041)
Supplement: oyag041_Supplementary_Data [file oyag041_supplementary_data.docx]

**Supplementary Table 1.** Summary of key prognostic models for patients with resected RCC

| Model | Risk Factors | N. of patients in validation cohort | Setting | Histology | Endpoint | c-index |
| --- | --- | --- | --- | --- | --- | --- |
| UISS | TNM, Fuhrman grade, ECOG PS | 4,202 | Surveillance | All | OS | 0.76-0.86 |
| SSIGN | TNM, tumor size, grade and necrosis | 3,600 | Surveillance | ccRCC only | CSS | 0.82-0.84 |
| Leibovich 2003 | TNM, tumor size, grade and necrosis | 1,671 | Surveillance | ccRCC only | RFS | 0.82 |
| GRANT | TNM, Fuhrman grade, age | 1,926 | ASSURE adjuvant trial | All | DFS, OS | 0.65 |

Abbreviations: **UISS**: University of California Los Angeles Integrated Staging System; **SSIGN**: Stage, Size, Grade, and Necrosis; **GRANT**: Grade, Age, Nodes, Tumor; **ASSURE**: Adjuvant Sorafenib or Sunitinib for Unfavorable RCC; **TNM**: Tumor-Node-Metastasis staging system; **ECOG PS**: Eastern Cooperative Oncology Group Performance Status; **ccRCC**: Clear Cell Renal Cell Carcinoma; **OS**: Overall Survival; **CSS**: Cancer-Specific Survival; **RFS**: Recurrence-Free Survival; **DFS**: Disease-Free Survival.

**Supplementary Fig. 1.** Consort Diagram


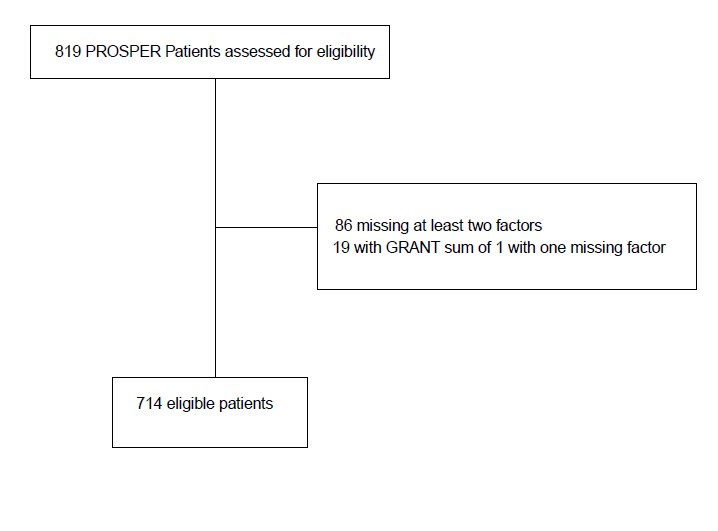


**Supplementary Fig. 2.** DCA plot for recurrence-free survival and GRANT risk groups.


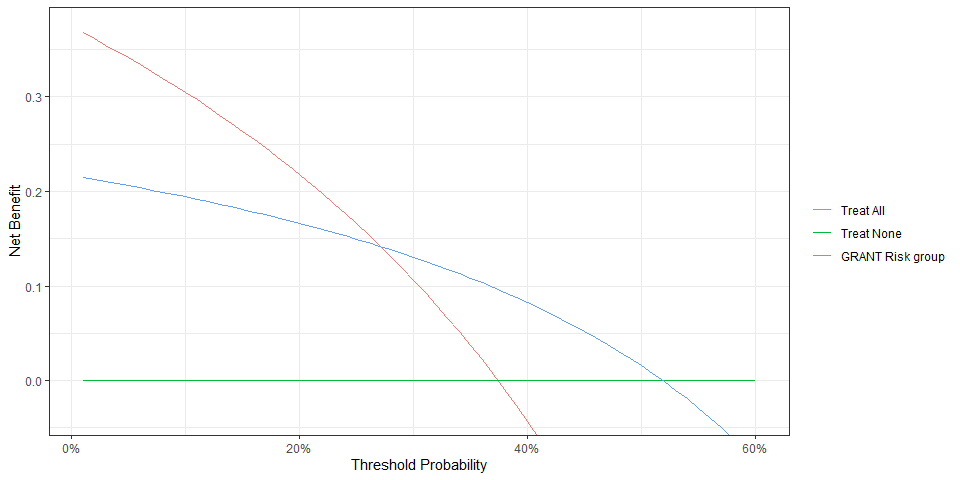


**Supplementary Table 2**. Subgroup univariable Cox regression models for recurrence-free survival by GRANT group and treatment arm, with interaction analysis (n = 709). *GRANT favorable group: score 0-1; GRANT unfavorable group: score 2-4 (1 point each for: age >60 years, tumor grade >2, pT-stage ≥pT3b and pN1).*

| **Arm** | **Number of event / total** | **Median [95% CI]**  **(months)** | **HR [95% CI]**  **(Nivolumab plus surgery/surgery only)** | ***p*-value** |
| --- | --- | --- | --- | --- |
| Favorable GRANT risk group (n = 413) | | | | |
| Nivolumab plus surgery arm | 33/205 | 61.1 [NA – NA] | 0.66 [0.42 – 1.05] | 0.075 |
| Surgery only arm | 45/208 | NA [NA – NA] |  |  |
| Unfavorable GRANT risk group (n = 296) | | | | |
| Nivolumab plus surgery arm | 58/134 | 39.1 [27.3 – NA] | 0.90 [0.64 – 1.28] | 0.56 |
| Surgery only arm | 69/162 | 36.9 [21.0 – NA] |  |  |
| Interaction analysis | | | | |
| GRANT risk group  Favorable vs. Unfavorable (ref) | | | 0.41 [0.28 – 0.60] | < **0.001** |
| Treatment arm  Nivolumab plus surgery vs. surgery only arm (ref) | | | 0.89 [0.63 – 1.27] | 0.53 |
| GRANT risk group*treatment arm | | | 0.75 [0.42 – 1.33] | 0.33 |

**Supplementary Fig. 3.** Kaplan-Meier curves of recurrence-free survival by treatment arm in the favorable (A) and unfavorable (B) GRANT group. *GRANT favorable group: score 0-1; GRANT unfavorable group: score 2-4 (1 point each for: age >60 years, tumor grade >2, pT-stage ≥pT3b and pN1).*


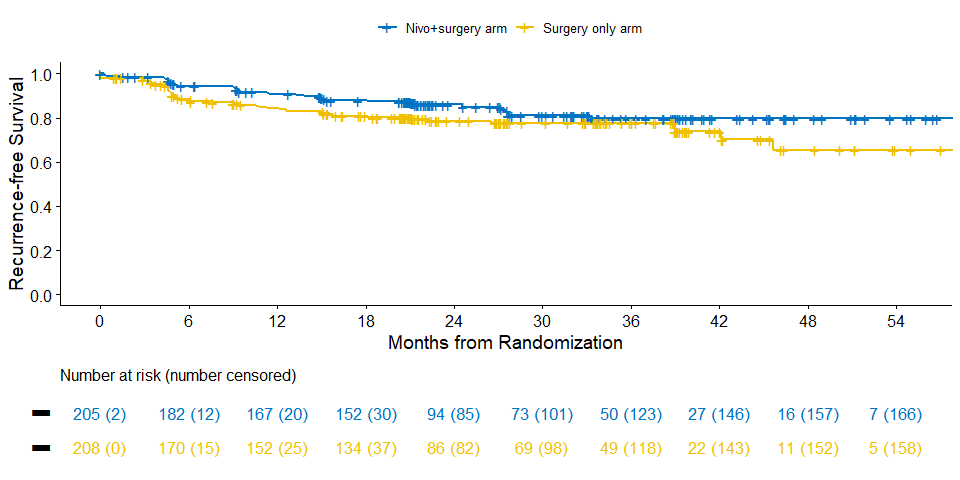


**A**


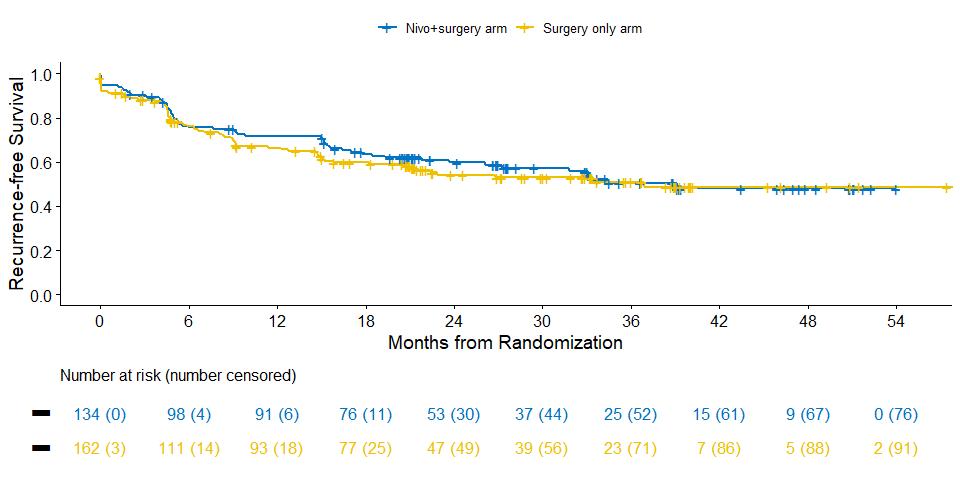


**B**

**Supplementary Fig. 4.** Kaplan-Meier curves of recurrence-free survival by GRANT groups in clear cell (A) and non-clear cell histology (B). *GRANT favorable group: score 0-1; GRANT unfavorable group: score 2-4 (1 point each for: age >60 years, tumor grade >2, pT-stage ≥pT3b and pN1).*


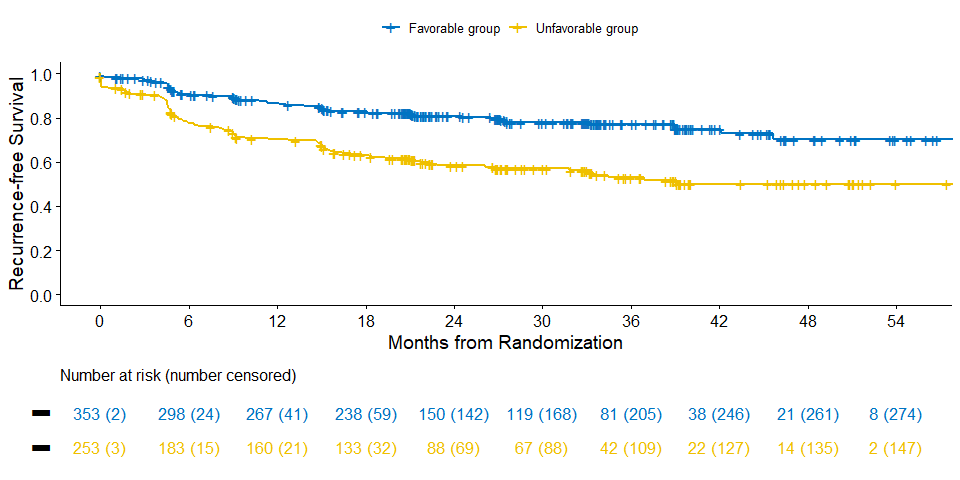


**A**


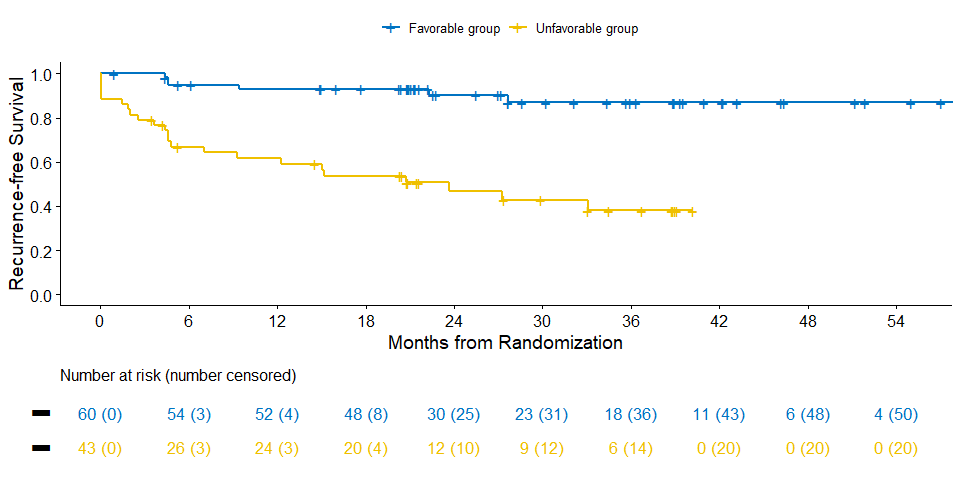


**B**

**Supplementary Table 3.** 2- and 3-year recurrence-free survival by GRANT group and histology subtype. *GRANT favorable group: score 0-1; GRANT unfavorable group: score 2-4 (1 point each for: age >60 years, tumor grade >2, pT-stage ≥pT3b and pN1).*

| **Group** | **2-year RFS [95% CI]**  **(%)** | **3-year RFS [95% CI]**  **(%)** |
| --- | --- | --- |
| Clear cell histology | | |
| Favorable | 80.9 [76.6 – 85.4] | 77.2 [72.3 – 82.4] |
| Unfavorable | 58.5 [52.3 – 65.3] | 53.0 [46.2 – 60.8] |
| Non-clear cell histology | | |
| Favorable | 90.3 [82.3 – 99.0] | 86.9 [77.2 – 97.9] |
| Unfavorable | 46.8 [33.1 – 66.2] | 38.2 [24.3 – 59.8] |

**Supplementary Fig. 5.** DCA plot of GRANT score for recurrence-free survival among clear cell (A) and non-clear cell histology (B)


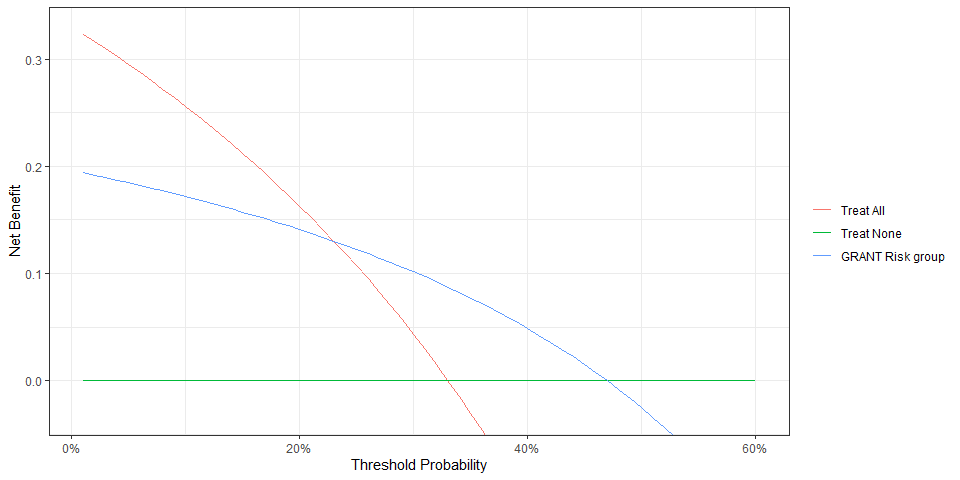


**A**


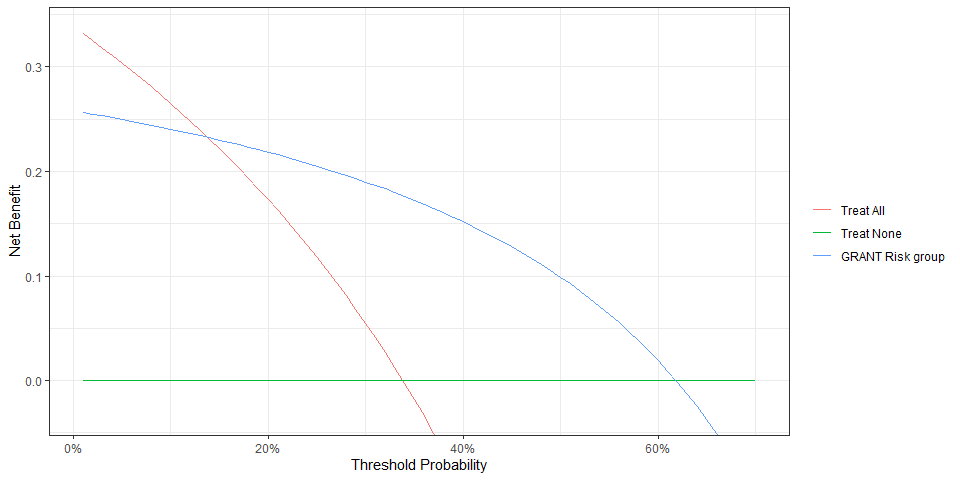


**B**

**Supplementary Fig. 6.** DCA plot for overall survival and GRANT risk groups


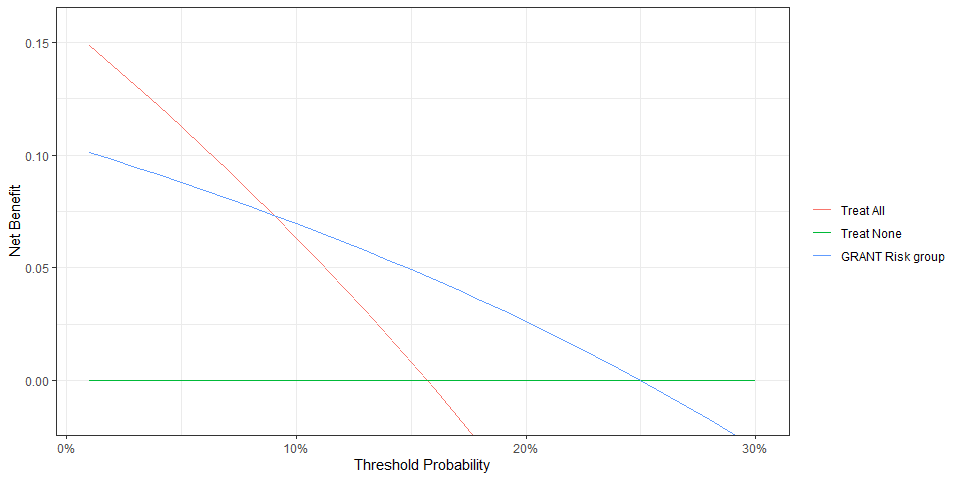


**Supplementary Table 4**. Subgroup univariable Cox regression models for overall survival by GRANT group and treatment arm, with interaction analysis (n = 714). *GRANT favorable group: score 0-1; GRANT unfavorable group: score 2-4 (1 point each for: age >60 years, tumor grade >2, pT-stage ≥pT3b and pN1).*

| **Arm** | **Number of event / total** | **Median [95% CI]**  **(months)** | **HR [95% CI]**  **(Nivolumab plus surgery/surgery only)** | **p-value** |
| --- | --- | --- | --- | --- |
| Favorable GRANT risk group (n = 416) | | | | |
| Nivolumab plus surgery arm | 10/208 | NA [NA – NA] | 1.00 [0.42 – 2.41] | > 0.99 |
| Surgery only arm | 10/208 | NA [NA – NA] |  |  |
| Unfavorable GRANT risk group (n = 298) | | | | |
| Nivolumab plus surgery arm | 29/134 | NA [59.5 – NA] | 1.20 [0.71 – 2.04] | 0.50 |
| Surgery only arm | 26/164 | NA [NA – NA] |  |  |
| Interaction analysis | | | | |
| GRANT risk group  Favorable vs. Unfavorable (ref) | | | 0.27 [0.13 – 0.57] | < **0.001** |
| Treatment arm  Nivolumab plus surgery vs. surgery only arm (ref) | | | 1.20 [0.71 – 2.04] | 0.50 |
| GRANT risk group*treatment arm | | | 0.84 [0.30 – 2.33] | 0.73 |

**Supplementary Fig. 7.** Kaplan-Meier curves of overall survival by treatment received in the favorable (A) and unfavorable (B) GRANT group. *GRANT favorable group: score 0-1; GRANT unfavorable group: score 2-4 (1 point each for: age >60 years, tumor grade >2, pT-stage ≥pT3b and pN1).*


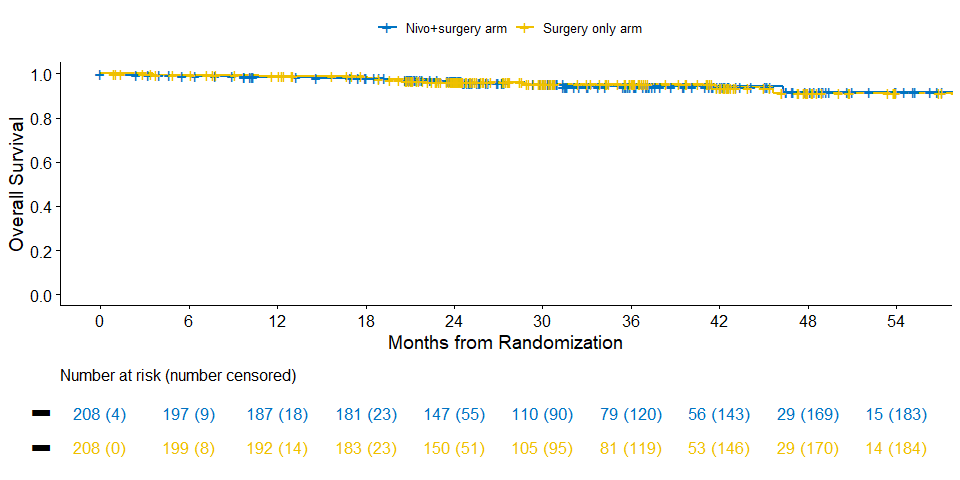


**A**


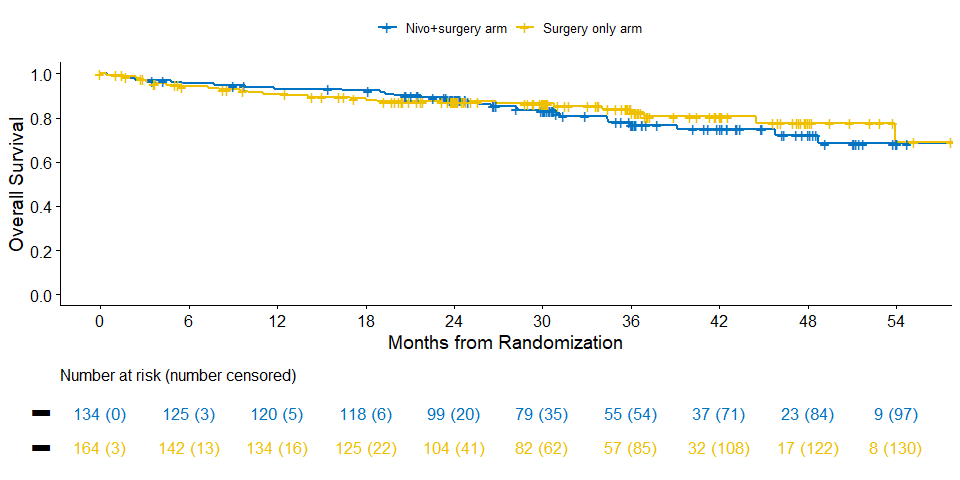


**B**

**Supplementary Table 5**. Subgroup univariable Cox regression models for overall survival by GRANT group and histology subtype, with interaction analysis (n = 709). *GRANT favorable group: score 0-1; GRANT unfavorable group: score 2-4 (1 point each for: age >60 years, tumor grade >2, pT-stage ≥pT3b and pN1).*

| **GRANT risk group** | **Number of event / total** | **Median**  **[95%CI]**  **(months)** | **HR**  **[95%CI]**  **(Favorable/unfavorable)** | ***p*-value** | ***C*-index**  **[95%CI]** |
| --- | --- | --- | --- | --- | --- |
| Clear cell histology (n = 606) | | | | | |
| Favorable | 17/353 | NA [NA – NA] | 0.29  [0.16 – 0.50] | < **0.001** | 0.64  [0.58 – 0.71] |
| Unfavorable | 42/253 | NA [59.5 – NA] |  |  |  |
| Non-clear cell histology (n = 103) | | | | | |
| Favorable | 3/60 | NA [NA – NA] | 0.14  [0.04 – 0.50] | < **0.001** | 0.74  [0.65 – 0.83] |
| Unfavorable | 12/43 | NA [44.5 – NA] |  |  |  |
| Interaction analysis | | | | | |
| GRANT risk group  Favorable vs. Unfavorable (ref) | | | 0.14 [0.04 – 0.50] | **0.003** |  |
| Histology subtype  Clear cell RCC vs. Non-clear cell RCC (ref) | | | 0.50 [0.27 – 0.96] | **0.037** |  |
| GRANT risk group*histology subtype | | | 2.02 [0.50 – 8.08] | 0.32 |  |

**Supplementary Fig. 8.** Kaplan-Meier curves of overall survival by GRANT groups in clear cell (A) and non-clear cell histology (B). *GRANT favorable group: score 0-1; GRANT unfavorable group: score 2-4 (1 point each for: age >60 years, tumor grade >2, pT-stage ≥pT3b and pN1).*


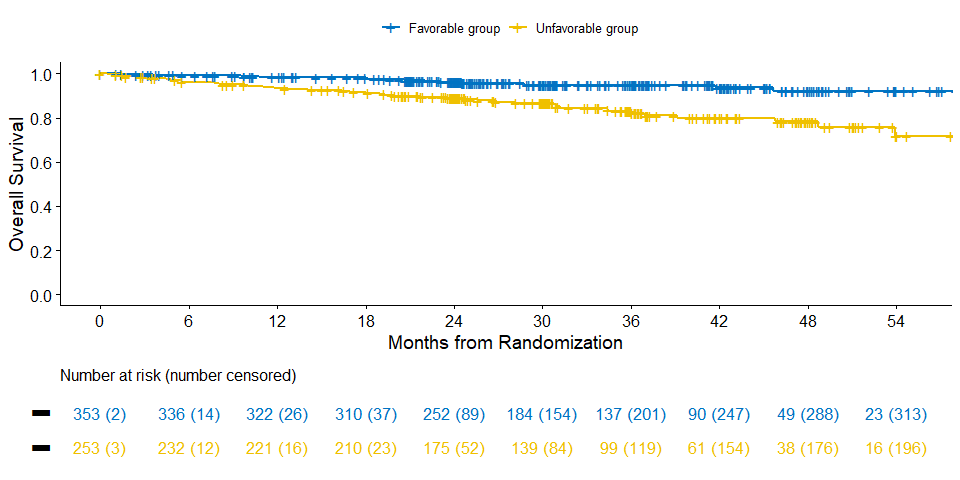


**A**


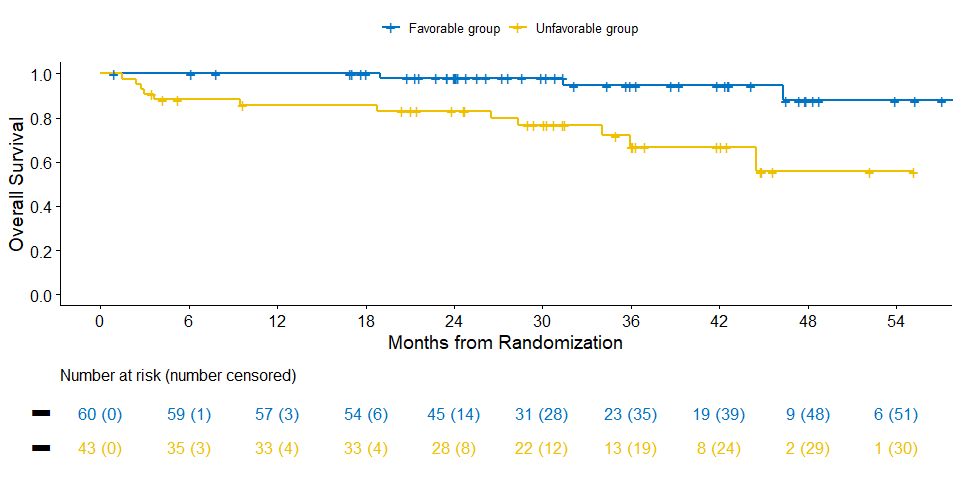


**B**

**Supplementary Table 6.** 2-year and 3-year overall survival by GRANT group and histology subtype. *GRANT favorable group: score 0-1; GRANT unfavorable group: score 2-4 (1 point each for: age >60 years, tumor grade >2, pT-stage ≥pT3b and pN1).*

| **Group** | **2-year [95% CI]**  **(%)** | **3-year OS [95% CI]**  **(%)** |
| --- | --- | --- |
| Clear cell histology | | |
| Favorable | 96.2 [94.1 – 98.4] | 94.8 [92.3 – 97.5] |
| Unfavorable | 89.0 [85.1 – 93.1] | 83.0 [77.9 – 88.5] |
| Non-clear cell histology | | |
| Favorable | 98.1 [94.5 – 100.0] | 94.6 [87.3 – 100.0] |
| Unfavorable | 83.2 [72.5 – 95.4] | 66.9 [51.5 – 86.8] |

**Supplementary Fig. 9.** DCA plot of GRANT score for overall survival among clear cell (A) and non-clear cell histology (B).


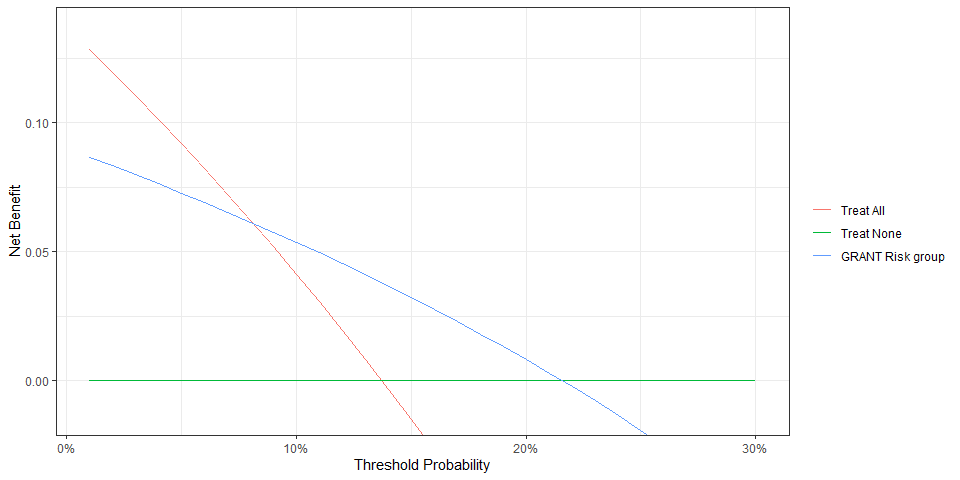


**A**


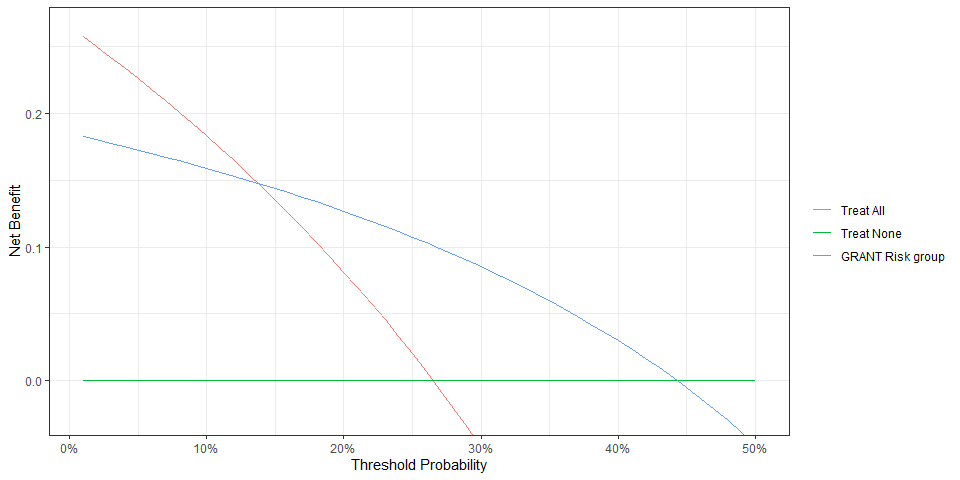


**B**
